# Clinical Profile and Outcome of Postthymectomy versus Non-Thymectomy Myasthenia Gravis Patients in the Philippine General Hospital: A 6-Year Retrospective Study

**DOI:** 10.3389/fneur.2016.00096

**Published:** 2016-06-21

**Authors:** Ranhel C. De Roxas, Marjorie Anne C. Bagnas, Jobelle Joyce Anne R. Baldonado, Jonathan P. Rivera, Artemio A. Roxas

**Affiliations:** ^1^Department of Neurosciences, Philippine General Hospital, Manila, Philippines; ^2^Department of Surgery, Philippine General Hospital, Manila, Philippines; ^3^Department of Pathology, Philippine General Hospital, Manila, Philippines

**Keywords:** myasthenia gravis, thymoma, non-thymomatous, thymectomy

## Abstract

**Background:**

Myasthenia gravis is an autoimmune neuromuscular disorder characterized by the production of abnormal autoantibodies directed against the receptors present in the neuromuscular junction. It has been the standard practice to offer thymectomy in all generalized myasthenia gravis patients despite the lack of robust evidence.

**Objectives:**

The objectives of this study are to describe the clinical profile and differentiate the clinical outcomes of thymectomy versus non-thymectomy and thymomatous versus non-thymomatous myasthenia gravis patients in the Philippine General Hospital.

**Methodology:**

Between 2009 and 2014, a total of 69 postthymectomy and 16 non-thymectomy patient records were successfully retrieved. The demographic characteristics, surgical approach, and histopathologic results were obtained. The clinical outcome after 6 months or 1 year-follow-up was also determined and grouped according to the following: (1) complete remission, (2) pharmacological remission, (3) no clinical change, (4) worsening symptoms, and (5) mortality.

**Results:**

Majority of the patients were females (68.0%) with a mean age of 39.8 years and a mean duration of myasthenic symptoms of 21 months. Using the Myasthenia Gravis Foundation of America classification, 54.1% of patients fell under Class II and 48.2% of them presented with generalized weakness. In this study, 60.8% of postthymectomy myasthenia gravis patients had either complete remission or pharmacologic remission compared with 12.5% among non-thymectomy patients (*p*-value <0.001). No significant difference in the clinical outcome was found between thymomatous and non-thymomatous myasthenia gravis after thymectomy (*p*-value = 0.29).

**Conclusion:**

This study showed that both thymomatous and non-thymomatous myasthenia gravis patients who underwent thymectomy had a higher incidence of complete stable remission and pharmacologic remission as compared with myasthenia gravis patients who did not undergo thymectomy.

## Background

Myasthenia gravis is an autoimmune neuromuscular disorder characterized by the production of abnormal autoantibodies directed against nicotinic acetylcholine receptors (AChR) present in the neuromuscular junctions. It presents with weakness of voluntary ocular, bulbar, and limb muscles, and in severe cases, death from difficulty of breathing (1, 2). In a large epidemiological study done within a 60-year period, the pooled incidence of myasthenia gravis is 5.3 per million person years with a prevalence rate of 15 times higher probably attributable to the good survival and low mortality of the disease (3).

The pathogenesis of myasthenia gravis is due to the presence of antibodies significantly interfering the neuromuscular junction. Antibodies against the AChR and the muscle specific kinase (MuSK) are the two major types of antibodies found (4). Anti-AChR is postulated to accelerate degradation of AChR, functionally block the acetylcholine binding sites, and cause complement-mediated damage of the postsynaptic membrane. On the other hand, anti-MuSK inhibits the clustering of AChR on the postsynaptic membrane causing the increased muscle fatigability (5).

The treatment of myasthenia gravis is geared toward improving the neuromuscular transmission, interfering with the antibodies, and modifying the natural history of the disease (5). Acetylcholineserase inhibitors, such as pyridostigmine, prolong the availability of acetylcholine in the neuromuscular junction and are regarded as the first-line treatment for myasthenia gravis (6). Steroids, usually prednisone, are added in the chronic therapeutic regimen of myasthenia gravis patients. Its effective immunosuppressive activity provides clinical benefit in about 80% of the patients (7). Several other immunosuppressive agents, such as azathioprine, cyclosporin A, cyclophosphamide, mycophenolate mofetil, and methotrexate, are used as an alternative or in combination with steroids; however, their efficacy are yet to be established by well-designed clinical trials. In some patients on whom rapid effect is warranted, intravenous immune globulin and plasmapheresis act as rapid immunotherapeutic options (8). It has been the practice to offer thymectomy in all myasthenia gravis patients, especially to those who are younger than 60 years old and with generalized symptoms, even if its efficacy in improving outcomes is still under study (9). Theoretically, thymectomy may eliminate the source of autoantigens, remove the B cell reservoir secreting antibodies, and correct the disturbance of immune regulation (10). In a study by Blalock et al., complete resolution of symptoms in thymomatous myasthenia gravis was first found in a 21-year-old female after thymectomy (11). In another study, there was also improvement even in non-thymomatous myasthenia gravis postthymectomy (12). Most studies have shown higher remission rates from 13 to 46% in symptomatic patients undergoing thymectomy compared with those who were treated only pharmacologically (13–15). However, the role of thymectomy is still uncertain and the available studies still have inconclusive results in the efficacy, indication, and timing for thymectomy between thymomatous and non-thymomatous myasthenia gravis (16, 17). In the Philippines, the present consensus recommends thymectomy as an option to increase the likelihood of remission between thymomatous and non-thymomatous myasthenia gravis patients until randomized controlled trials become available (18).

## Rationale and Objectives

The primary objective of this study is to compare the clinical outcome of myasthenia gravis patients who underwent thymectomy versus those who did not undergo thymectomy. This study also aims to describe the clinical profile of patients with myasthenia gravis and to determine the clinical outcome of thymomatous versus non-thymomatous myasthenia gravis after thymectomy. Knowing their outcomes could help us in the selection of patients whom thymectomy could be appropriately offered in the future.

## Methodology

### Study Design and Study Population

This is a 6-year retrospective cohort study from January 2009 to December 2014. The medical records of all myasthenia gravis patients admitted in the charity wards were obtained and reviewed. The list of 149 patients who underwent thymectomy was obtained from the database of the Thoracic and Cardiovascular Surgery section, whereas the list of 27 myasthenia gravis patients who did not undergo thymectomy was obtained from the Adult Neurology section electronic census of charity inpatients. The lists were submitted to the Medical Records Section for retrieval. All recoverable medical records of adult myasthenia gravis patients aged more than 18 years of age were included in the study. Missing records were excluded.

### Data Collection

The age, sex, comorbidities, onset and duration of symptoms, characteristics of symptoms, and medications were recorded. The myasthenia gravis severity was evaluated using the Myasthenia Gravis Foundation of America (MGFA) classification. The clinical outcome of patients with myasthenia gravis who did not undergo thymectomy was reviewed after 6 months or 1 year of starting pharmacologic treatment, depending on the available follow-up data. The clinical outcome was grouped according to the following: (1) complete remission, (2) pharmacological remission, (3) no clinical change, (4) worsening symptoms, and (5) mortality. The preoperative characteristics, surgical approach, histopathologic results, and clinical outcomes on the sixth month or 1-year postoperative follow-up of those who underwent thymectomy were also reviewed. All final histopathologic results were obtained from the Surgical Pathology Unit of the Department of Pathology. Patients who had no follow-up data were contacted through voice call to determine their clinical outcome.

### Study Analysis

These data were encoded using Microsoft Excel and analyzed using the Microsoft Excel Data analysis toolpack and the Epi-Info 7.0 program. The difference in the clinical outcome of postthymectomy patients versus patients who did not undergo thymectomy was analyzed using the Fisher’s exact test. The difference between thymomatous and non-thymomatous groups among postthymectomy patients was also analyzed using the Fisher’s exact test.

### Definition of Terms

(1) Prolonged intubation – defined as difficulty weaning off the mechanical ventilator after ≥14 days.
(2) Complete stable remission – corresponds to the state when the patient does not have symptoms anymore even with no medications for a period of at least 6 months.
(3) Pharmacologic remission – corresponds to at least 50% decrease in the need of medications to control the symptoms of myasthenia gravis.
(4) Worse symptoms – defined as any amount of increase in the need for medications in order to control the myasthenic symptoms.
(5) Good response – corresponds to either complete stable remission or pharmacologic remission.
(6) Poor response – corresponds to whether the patient had no clinical change, worse symptoms, or died within the follow-up period.

### Ethical Considerations

This study was submitted and approved by the University of the Philippines Manila Research Ethics Board Panel. The data collection commenced upon approval from the panel and the anonymity of subjects, as well as the confidentiality of the data obtained, was assured by the researcher.

## Results

A total of 134 out of the 176 patient records (71.1%) were successfully retrieved from the Medical Records section. Among those who underwent thymectomy, 118 out of the 149 patient records were obtained. Only 69 (58.5%) patients were found to have myasthenia gravis, and the other patients underwent thymectomy for other indications (Table 1). Out of the 27 myasthenia gravis inpatients who did not undergo thymectomy, only 16 patient records were retrieved. The 42 missing records from both lists were excluded in the study.

**Table 1:** **Other indications for thymectomy other than myasthenia gravis**.

	**Number (%)**
Thymoma without myasthenia gravis	35 (29.7)
Metastasis	3 (2.5)
Germ cell tumor	5 (4.2)
Tuberculosis	1 (0.8)
Others (cysts, lymphoma)	5 (4.2)

The age of all the myasthenia gravis patients included in this study ranges from 18 to 69 years with a mean age of 39.8 years in both the postthymectomy and non-thymectomy patients. Majority of the patients were females (68.0%). It was also found that 86.0 and 66.0% of the female and male patients, respectively, had symptoms before the age of 50 years. The mean duration of myasthenia gravis symptoms was 21 months where most of the patients presented with generalized weakness (48.2%), followed by ocular symptoms reported as ptosis and/or diplopia in 47.1%, and bulbar symptoms described as dysphagia in 34.1% of the patients. Fifteen patients (17.6%) had pure ocular symptoms while only three (3.5%) were presented with dominant bulbar symptoms. Using the MGFA classification, more than half of the patients had mild symptoms (IIa = 16.5%, IIb = 37.6%), while 14 (IIIa = 3.5%, IIIb = 12.9%) and 3 (IVa = 0%, IVb = 3.5%) had moderate and severe symptoms, respectively. Only a small proportion of the patients (15.3%) necessitated prolonged intubation usually due to complications of pneumonia. Pyridostigmine and prednisone were the mainstay therapy for 70.6% of the patients. Table 2 shows the baseline characteristics of all the myasthenia gravis patients included in the study.

**Table 2:** **Baseline characteristics of myasthenia gravis patients included in the study**.

**Baseline characteristics**	**Postthymectomy (*n*** **= 69)**	**Non-thymectomy (*n*** **= 16)**	**Total (*n*** **= 85)**
Age	40.2 ± 13.1	38.1 ± 12.5	39.8 ± 12.9
Gender
Male	23 (33.3)	4 (25.0)	27 (32.0)
Female	46 (66.6)	12 (75)	58 (68.0)
Age at presentation	38.4 ± 13.8	35.4 ± 12.4	37.6 ± 13.5
<30 years old	19 (27.5)	6 (37.5)	25 (29.4)
31–50 years old	27 (39.1)	6 (37.5)	33 (38.8)
>50 years old	23 (33.3)	4 (20.0)	27 (31.8)
Comorbidities
None	38 (55.1)	4 (25.0)	42 (49.4)
Pneumonia	16 (23.2)	10 (62.5)	26 (30.6)
Goiter	5 (7.2)	2 (12.5)	7 (8.2)
Hypertension	8 (11.6)	3 (18.7)	11 (12.9)
Diabetes mellitus	3 (4.3)	1 (6.2)	4 (4.7)
Others	3 (4.3)	5 (31.2)	8 (9.4)
Myasthenia gravis symptoms
Ocular	34 (49.3)	6 (37.5)	40 (47.1)
Bulbar	24 (34.8)	5 (31.2)	29 (34.1)
Generalized	31 (44.9)	10 (62.5)	41 (48.2)
Duration of symptoms (range: 0.3–144.0 months)	21.2 ± 26.5	22.5 ± 28.2	21.5 ± 26.7
Pharmacologic treatment
Pyridostigmine	12 (17.4)	1 (6.2)	13 (15.3)
Pyridostigmine + prednisone	47 (68.1)	13 (81.2)	60 (70.6)
Pyridostigmine + azathioprine	2 (2.9)	1 (6.2)	3 (3.5)
≥3 drugs	8 (11.6)	1 (6.2)	9 (10.6)
MGFA classification
I	14 (20.3)	1 (6.2)	15 (17.6)
II	39 (56.5)	7 (43.7)	46 (54.1)
III	11 (15.9)	3 (18.7)	14 (16.5)
IV	3 (4.3)	0 (0.0)	3 (3.5)
V	2 (2.9)	5 (31.2)	7 (8.2)
Prolonged intubation
Yes	9 (13.0)	4 (25.0)	13 (15.3)
No	60 (87.0)	12 (75.0)	72 (84.7)

Three out of the 85 included patients had no follow-up record. In this study, it was found that 60.8% of postthymectomy myasthenia gravis patients had good outcome compared with 12.5% of the non-thymectomy patients after at least 6 months of follow-up (*p*-value <0.001). Among the 67 postthymectomy patients, those who presented with good outcome mostly presented with mild symptoms (83.3%) before thymectomy. Even most of the patients with pure ocular myasthenia gravis (60.0%) were found to have clinical improvement after thymectomy. Majority of the patients with poor outcome had onset of symptoms before the age of 50 years (*p*-value = 0.55) and four of them died because of comorbid complications such as pneumonia, pulmonary embolism, and arrhythmia. Among those who did not undergo thymectomy, 81.2% of patients had poor outcome within 6 months to 1 year of follow-up and none of them achieved complete myasthenia gravis remission. Table 3 summarizes the clinical outcome of the myasthenia gravis patients included in the study.

**Table 3:** **Clinical outcomes of postthymectomy patients versus those who did not undergo thymectomy after 6 months and/or 1 year follow-up**.

	**Postthymectomy patients (*n*** **= 69)**	**%**	**Non-thymectomy patients (*n*** **= 16)**	**%**
Complete stable remission	10	14.5	0	0.0
Pharmacological remission	32	46.3	2	12.5
No clinical change	12	17.4	6	37.5
Worse symptoms	9	13.0	2	12.5
Mortality	4	5.8	5	31.2
No follow-up record	2	2.96	1	6.2

Among the postthymectomy patients, majority underwent median sternotomy (86.2%) while only a small proportion of patients underwent video-assisted thoracoscopic thymectomy (13.8%). It was also found that 29 (42.0%) were thymomatous by histopathologic diagnosis, most of which was classified as Thymoma Type B (18.7%) based on the World Health Organization (WHO) classification of thymoma. Another 29 (42.0%) were non-thymomatous, majority of which were thymic lymphoproliferative tumor (Table 4). A total of 11 patients had no histopathologic record available from the Surgical Histopathologic Unit of the Philippine General Hospital. Non-thymomatous myasthenia gravis patients were found to have longer duration of preoperative symptoms (33.8 months) but tend to have shorter hospital stay (8.4 days) compared with thymomatous myasthenia gravis. Moreover, non-thymomatous myasthenia gravis had higher percentage with good outcome (62.1%, *p*-value = 0.29) compared with thymomatous myasthenia gravis (51.7%) and tended to have shorter duration of hospital stay ranging from 1 to 34 days (Table 5).

**Table 4:** **Histopathologic diagnosis of postthymectomy patients**.

**Histopathologic results (*n*** **= 69)**	**Number (%)**
Thymic hyperplasia	17 (24.6)
Thymoma
Type A	6 (8.7)
Type AB	6 (8.7)
Type B1	4 (5.8)
Type B2	2 (2.8)
Type B3	7 (10.1)
Type C	4 (5.8)
Others	8 (11.6)
Histologically unremarkable thymic tissue	4 (5.8)
No histopathologic results	11 (15.9)

**Table 5:** **Clinical outcomes of thymomatous versus non-thymomatous myasthenia gravis after 6 months and/or 1 year postthymectomy**.

	**Thymomatous (*n*** **= 29)**	**%**	**Non-thymomatous (*n*** **= 29)**	**%**
Age
<30 years old	2	6.9	16	55.2
30–50 years old	20	69.0	8	27.6
>50 years old	7	24.1	5	17.2
Surgical approach
Median sternotomy	25	86.2	25	86.2
Video-assisted thoracoscopy	4	13.8	4	13.8
Preoperative duration of symptoms	18.3 months (range: 1–60 months)		33.8 months (range: 0.3–144 months)	
Duration of confinement	15.8 days (range: 2–85 days)		8.4 days (range: 1–34 days)
Good outcome	15	51.7	18	62.1
Poor outcome	13	44.8	10	34.5

## Discussion

This study determined the clinical profile and outcome of myasthenia patients in our institution. Most of the myasthenia patients were young females aged <50 years old. This finding was also observed in a recent epidemiological study in Taiwan consisting of 5,211 patients where 60.7% were females in the 15–54 age group (19). The higher percentage of females <50 years old and males >50 years old may be reflective of the usual bimodal pattern of myasthenia gravis where it first peaks in the third decade among females and then in the sixth decade among males (20). The MGFA classification was designed to uniformly classify myasthenia gravis despite the subjective nature of the disease and is usually utilized to determine its severity (21). Around 54.0% of the patients fell under Class II corresponding to mild symptoms. Also, about half of the patients presented with generalized weakness. This is so probably because most of the patients sought consult when the generalization of myasthenia gravis sets in, which usually occurs within the first 2 years of the disease (22). Even if majority of patients initially present with ocular symptoms, studies have shown that approximately 50% of patients progress to generalized myasthenia gravis in their lifetime (23). In 70.6% of the patients, the combination of pyridostigmine and prednisone were the therapeutic mainstay. Although previous studies have shown that most myasthenia gravis patients were solely maintained on anticholinesterase inhibitor and only about one-fourth of patients necessitated combination with steroids, it was found to be more sufficient to give combination therapy especially in generalized myasthenia gravis (24, 25).

Thymectomy is currently a recommended treatment option in myasthenia gravis even in the absence of randomized controlled trials showing its benefits (18). Different surgical approaches are available, ranging from extended transsternal thymectomy to minimally invasive video-assisted thoracosopic surgery to the newly developed robotic surgery (26). In this study, most patients underwent median sternotomy, and only a small proportion underwent video-assisted thoracoscopic surgery. Yet, none underwent robotic surgery. In a study by Zahid et al., video-assisted thoracoscopic surgery was found to have no statistically significant difference in terms of remission and recurrence rates compared with median sternotomy. However, patients were found to have shorter hospital stay, lesser blood loss, and greater cosmetic satisfaction (27). An 18-month lag from the time of symptom development to surgery was found among thymomatous myasthenia gravis, which is shorter than that of non-thymomatous myasthenia gravis. This duration was longer than that in literature, and this difference may suggest that thymectomy is probably not readily offered in non-thymomatous myasthenia gravis until it was observed that symptoms does not resolve even with medications (24).

In this study, postthymectomy patients were shown to have statistically significant higher percentage with complete stable remission and pharmacologic remission compared with patients who did not undergo surgery. This finding is in line with that of previous literature where remission or reduction in medication was found in approximately 70% of the myasthenia gravis patients after thymectomy (28). This was also elucidated in a previous local study consisting of 10 myasthenia gravis patients who had clinical improvement after thymectomy (29). Also, it was found that myasthenia gravis with mild severity, including those with pure ocular involvement, seems to have better outcome and higher remission rate, which is consistent with previous studies (24, 30–32). Poor outcome after thymectomy was seen more frequently in patients whose myasthenia gravis symptoms occurred before the age of 50 years. This finding supports an earlier study that thymectomy may be done and even be of benefit in patients with late-onset myasthenia gravis (age >50 years) (33).

This study also showed that there is no difference in the clinical outcome of thymomatous myasthenia gravis as compared with that of non-thymomatous in histology. Thymomatous myasthenia is an absolute indication for thymectomy with an approximately 50% increase in survival at 5 years (34). In this study, thymoma accounts for 34.1% of myasthenia gravis patients, and this is higher from previous literature of 15% (35). Despite the established benefit of thymectomy in thymomatous myasthenia gravis, there are studies showing an even better clinical outcome in non-thymomatous myasthenia gravis as compared with thymomatous myasthenia gravis after thymectomy (28, 35, 36). This is postulated because of the presence of titin and another autoantibody to the RyR channel of the sarcoplasmic reticulum in patients with thymoma, which is associated to an increase of the severity of myasthenia gravis (37).

## Limitations

This study had several limitations. Only 71% of the patient records were obtained and this relatively low retrieval rate was because of the manual filing in the Medical Records section, which is prone to chart loss and misplacement. The missing records may actually change the results of this study. Moreover, the selection of patients was based on the available patient database in our institution. Since only inpatients were included, this means that they were admitted either because they underwent surgery or because they were sick requiring medical management. This may also be the reason why majority of patients already presented with generalized symptoms. Also, the determination of the patient clinical outcomes were only based on record review and voice call, hence, underreporting was a possibility. Lastly, a definitive conclusion cannot be made between the groups compared in this study because the baseline characteristics of the patients were not similar throughout the duration of the study.

## Conclusion

In summary, this study showed that both thymomatous and non-thymomatous myasthenia gravis patients who underwent thymectomy had a higher prevalence of complete stable remission and pharmacologic remission as compared with myasthenia gravis patients who did not undergo thymectomy. Although a definite conclusion cannot be made due to the inherent limitations of this study, it may just be acceptable to offer this option especially when generalized weakness is already evident.

## Recommendations

The authors strongly recommend an electronic myasthenia gravis patient registry in our institution and even in the national level in order to ensure complete inclusion of patients in future studies. Also, a study including outpatients is highly recommended, so that a more generalizable picture of myasthenia gravis patients in the local setting may be obtained. A long-term follow-up is also recommended.

## Author Contributions

RR primary author, manuscript writing; AR adviser, protocol development; MB protocol development; JR data collection.

All authors have made substantial, direct and intellectual contribution to the work, and approved it for publication.

## Conflict of Interest Statement

The authors declare that the research was conducted in the absence of any commercial or financial relationships that could be construed as a potential conflict of interest.
